# Steroidogenesis in the adrenal dysfunction of critical illness: impact of etomidate

**DOI:** 10.1186/cc11415

**Published:** 2012-07-10

**Authors:** Nienke Molenaar, Ronald M Bijkerk, Albertus Beishuizen, Christel M Hempen, Margriet FC de Jong, Istvan Vermes, Gertjan van der Sluijs Veer, Armand RJ Girbes, AB Johan Groeneveld

**Affiliations:** 1 The Department of Intensive Care, VU University Medical Center, De Boelelaan 1117, Amsterdam, 1081 HV, The Netherlands; 2 The Department of Clinical Chemistry, Medical Spectrum Twente Hospital, P.O. Box 50000, Enschede, The Netherlands

## Abstract

**Introduction:**

This study was aimed at characterizing basal and adrenocorticotropic hormone (ACTH)-induced steroidogenesis in sepsis and nonsepsis patients with a suspicion of critical illness-related corticosteroid insufficiency (CIRCI), taking the use of etomidate-inhibiting 11β-hydroxylase into account.

**Method:**

This was a prospective study in a mixed surgical/medical intensive care unit (ICU) of a university hospital. The patients were 62 critically ill patients with a clinical suspicion of CIRCI. The patients underwent a 250-μg ACTH test (*n *= 67). ACTH, adrenal steroids, substrates, and precursors (modified tandem mass spectrometry) also were measured. Clinical characteristics including use of etomidate to facilitate intubation (*n *= 14 within 72 hours of ACTH testing) were recorded.

**Results:**

At the time of ACTH testing, patients had septic (*n *= 43) or nonseptic critical illness (*n *= 24). Baseline cortisol directly related to sepsis and endogenous ACTH, independent of etomidate use. Etomidate was associated with a lower baseline cortisol and cortisol/11β-deoxycortisol ratio as well as higher 11β-deoxycortisol, reflecting greater 11β-hydroxylase inhibition in nonsepsis than in sepsis. Cortisol increases < 250 m*M *in exogenous ACTH were associated with relatively low baseline (HDL-) cholesterol, and high endogenous ACTH with low cortisol/ACTH ratio, independent of etomidate. Although cortisol increases with exogenous ACTH, levels were lower in sepsis than in nonsepsis patients, and etomidate was associated with diminished increases in cortisol with exogenous ACTH, so that its use increased, albeit nonsignificantly, low cortisol increases to exogenous ACTH from 38% to 57%, in both conditions.

**Conclusions:**

A single dose of etomidate may attenuate stimulated more than basal cortisol synthesis. However, it may only partly contribute, particularly in the stressed sepsis patient, to the adrenal dysfunction of CIRCI, in addition to substrate deficiency.

## Introduction

**Figure 1:**
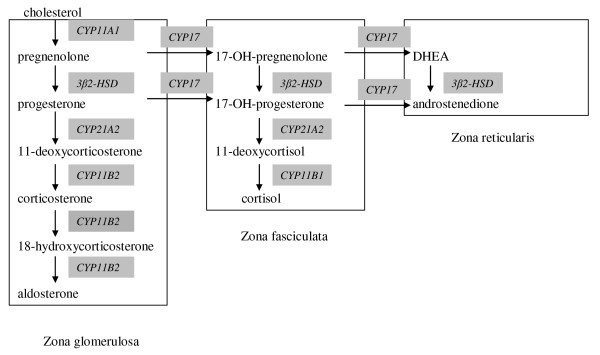
**Schematic presentation of steroidogenesis**. 3BHSD, 3-hydroxysteroid-dehydrogenase; CYP11B1, 11ß-hydroxylase; CYP11B2, 11 and 18-hydroxylase; CYP17, 17-hydroxylase and 17,20-hydroxylase; CYP21, 21-hydroxylase.

Activation of the hypothalamic-pituitary-adrenal (HPA) axis serves to adapt the organism to stress during critical illness, which may be differently regulated in sepsis and nonsepsis [1-7]. Particularly in septic shock, elevated levels of cortisol may be too low for the high level of stress, as compared with those in nonseptic critical illness. This may be associated with a diminished adrenal responsiveness to additional stress, reflected by a diminished cortisol response to exogenous adrenocorticotropic hormone (ACTH) [2,5,6]. Adrenal dysfunction may thereby, together with tissue cortisol resistance, contribute to critical illness-related corticosteroid insufficiency (CIRCI) and subsequent attributable morbidity and mortality. The diagnosis of CIRCI and treatment with moderate doses of hydrocortisone, particularly in septic shock, remain highly controversial [2,5,6,8-11]. Mechanisms of adrenal dysfunction, particularly in the course of sepsis and shock, may include impaired availability or cleaving of the substrate (HDL-)cholesterol and impaired activity of steroidogenic enzymes, limiting an adequate adrenal stress (ACTH) response [2,6,12-15] (see Figure 1). Impairment of cortisol secretion at baseline or with exogenous ACTH may also develop when (even a single dose of) etomidate, a known inhibitor of 11β-hydroxylase promoting conversion of 11β-deoxycortisol to cortisol, is used to facilitate intubation [2,4,6,16-29]. Indeed, cortisol precursors such as 11β-deoxycortisol and its ratio to cortisol have been studied to characterize steroidogenesis and thereby, occasionally, the role of etomidate and resultant 11β-hydroxylase inhibition in the adrenal dysfunction of CIRCI [16-20,23].

Hyperreninemic hypoaldosteronism also may develop in the critically ill [16,18,30,31]. This may contribute to hypotension and underlie the rationale for treatment of CIRCI with fludrocortisone, because inhibition of 11β-hydroxylase increases levels of 11β-deoxycorticosterone by inhibiting conversion to corticosterone and from there to aldosterone [2,16,18-20,30,31]. Hence, the adrenal dysfunction of CIRCI either may directly result from etomidate use [7,23,27,29,32], or etomidate may be only a risk factor [2,4,9,20-22,25,28]. The importance of prior etomidate for adrenal dysfunction, depending on underlying disease, and its harmful sequelae thus remain highly controversial [4,5,9,16,20-26,28,29,32,33]. During the adrenal dysfunction of CIRCI, impaired cortisol synthesis may also occur upstream of the final 11β-hydroxylase steps, thereby potentially attenuating an increase in 11β-deoxycortisol but not in circulating ACTH via feedback, as during 11β-hydroxylase inhibition [3,18]. Decreased 21-hydroxylase activity, increasing progesterone and 17-OH-progesterone, is a potential mechanism [20]. Decreased 3-hydroxysteroid dehydrogenase activity would result in decreased progesterone and 17-OH-progesterone and increased dehydroepiandrosterone(-sulfate) (DHEA(S)) relative to androstenedione levels, even though DHEA may be increased and DHEAS decreased in critical illness [34,35].

We analyzed adrenal steroidogenesis separately in sepsis and nonsepsis critically ill patients with suspected CIRCI, intubated with or without administration of etomidate, in response to endogenous or exogenous ACTH and on the basis of circulating hormones and precursors. The aim was to characterize adrenal dysfunction with the hypothesis that use of etomidate has a major impact on its occurrence and assessment.

## Materials and methods

### Patient population and study design

This prospective observational study was carried out in a mixed surgical/medical intensive care unit (ICU) of a university hospital from December 2004 to March 2007. Dutch legislation waives the need for informed consent as the ACTH test is routinely performed in the unit for clinical reasons, no extra blood is drawn for this study on precursors, and the provided results are treated anonymously. We included critically ill patients older than 18 years, admitted to the ICU, and being suspected of CIRCI. Suspicion of CIRCI was based on > 6 hours hypotension (< 100 mm Hg systolic) requiring repeated fluid challenges and/or vasopressor/inotropic treatment in the ICU. Patients were excluded if they had a history of HPA axis disease and if they had taken glucocorticosteroids in the preceding 24 hours. Patients were otherwise treated by attending intensivists in rotating shifts, in this 30-bed unit, admitting about 1,500 patients annually. Patients were admitted either after surgery or from medical wards. In our hospital, etomidate is used to facilitate intubation in the operating/recovery room or ICU, at a dose of 0.2 to 0.4 mg/kg; propofol (1 to 1.5 mg/kg) and/or midazolam (5 to 15 mg) are used as alternatives, at the discretion of the treating physician.

### Data collection

On the day of the ACTH test, the following parameters were recorded: time from ICU admission, age and sex, common clinical conditions at admission according to the international classification of disease-10 definitions and severity of illness, as assessed by the APACHE II score. Sepsis was defined as the presence of systemic inflammatory response syndrome (SIRS) with a clinical source of infection and a positive microbiologic local (urine, trachea, or other) and/or blood culture. SIRS was defined as two or more of the following criteria: a temperature > 38°C or < 36°C, a leukocyte count > 12 or < 4 × 10^9^/L, a heart rate > 90 beats per minute, and a respiratory rate > 20 breaths per minute, or arterial PCO_2 _< 32 mm Hg or the presence of mechanical ventilation. For laboratory measurements, blood was sampled before and 30 and 60 minutes after intravenous injection of 250 μg of synthetic ACTH (Synacthen). Blood was sampled in heparinized syringes, centrifuged at 3,000 rpm, and plasma was stored at -80°C until assayed. A low cortisol increase to ACTH was defined according to a cutoff level of 250 n*M *[1,2,4,5,7-9,11,14,23,33]. Interventions including type and doses of vasopressor/inotropics, use of etomidate in the preceding 72 hours, need for mechanical ventilation, and renal replacement therapy at the time of the ACTH test were recorded. Administration of phenytoin and fluconazole was recorded. After the day of the ACTH test, the following data were recorded: outcome until day 28 of ICU or hospital stay, outcome in the ICU, and length of ICU stay. Five patients (of whom three had sepsis) were tested more than once, at least 48 hours after discontinuation of hydrocortisone therapy and more than 72 hours after etomidate use, if any.

### Assays

Steroid precursor hormone levels were determined with help of tandem mass spectrometry (Applied Biosystems Q trap 3200) slightly modified after the previous description [36]. Reference values are shown in Table 1. The procedure was as follows. To 1 ml of sample, standard or quality control sample 150 μl of internal standard (cortisol-d4 and 17α-hydroxyprogesterone-d8) solution was added and deproteinized with 3 ml acetonitrile while vortexing. After centrifugation (20 minutes, 18°C, 3,000 rpm), the supernatant was evaporated at 58°C under a constant flow of nitrogen. The components were resolved in 1 ml methanol-water (1:3) by vortexing. The solution was further purified by solid-phase extraction (SPE) by using Polarplus Octadecyl C18 columns from JT Baker. The sample was transferred to the conditioned SPE columns, washed with 2 ml distilled water (AD), and eluted with 3 ml acetone. The eluent was evaporated, resolved with 100 L methanol-water (1:3), and transferred to high-performance liquid chromatography (HPLC) vials. For the liquid chromatography-mass spectrometry (LC-MS) measurements, a Shimadzu HPLC system consisting of two LC-20AD pumps, an SIL-20AC autosampler, a CTO-20A column oven, and a CBM-20A controller unit was coupled to a Q-Trap 3200 mass spectrometer from Applied Biosystems. All MS investigations were carried out with an atmospheric pressure photoionization source, which was operated in both positive and negative ion modes under multiple-reaction monitoring (MRM) conditions. Other laboratory measurements included total cholesterol (*n *< 6.5 m*M*) and HDL cholesterol (*n *> 0.9 m*M*) determined with enzymatic colorimetric assay (Modular P analytics; Roche Diagnostics, Mannheim, Germany). ACTH was determined with an immunometric assay, and CBG, with radioimmunoassay (Immulite 2500; Siemens Medical Diagnostic Solutions, Deerfield, IL, USA, and BioSource, Nivelles, Belgium; *n *30-54 mg/L). Cortisol was determined with competitive immunoassay (Centaur; Siemens Medical Diagnostic Solutions) as described before [5,9,11].

**Table 1:** Reference hormone values for adults

ACTH		< 9	p*M*
Progesterone (nonluteal phase)		< 5	n*M*
Corticosterone		< 30.0	n*M*
Aldosterone		< 5.0	n*M*
17-OH-progesterone		< 15.0	n*M*
11β-deoxycortisol		< 11.0	n*M*
Cortisol		150-600	n*M*
DHEA	< 60 years	1.0-25.0	μ*M*
	≥ 60 years	0.5-8.0	μ*M*
DHEAS		2.0-17.0	n*M*
Androstenedione		2.0-15.0	n*M*

ACTH, adrenocorticotropic hormone; DHEA(S), dehydroepiandrosterone (sulfate).

### Statistical analysis

The Fisher Exact test was used for categoric variables, and the Mann-Whitney *U *test, for continuous variables, because most data were distributed nonnormally (Kolmogorov-Smirnov test, *P *< 0.05). Generalized estimating equations (GEEs) were performed, taking repeated measurements in the same patients into account, to evaluate the effect of the underlying condition, use of etomidate, and their first-order interaction, after logarithmic transformation to normalize distributions. For correlations, the Pearson linear *r *was used. A two-sided *P *< 0.05 is considered to indicate statistical significance, and exact *P *values are given, unless < 0.001. Data are expressed as median (interquartile range).

## Results

### Patient characteristics

Table 2 shows patient characteristics on admission according to etomidate treatment. Twenty-one percent of patients had received etomidate for intubation within 72 hours before blood sampling and an ACTH test. No group differences were found, except for more-frequent respiratory failure as a reason for admission in the etomidate group. Mortality was unaffected by etomidate. Of note, most patients had received hydrocortisone.

**Table 2:** Patient characteristics

	**Etomidate** ***n *= 14**	**No etomidate** ***n *= 48**	***P***
Age, years	66 (22)	67 (17)	0.36
Sex, m/f	8 (57)/6 (43)	26 (54)/22 (46)	1.0
Admission syndromes^a^			
Trauma and postoperative	4(29)	19 (40)	0.54
Cardiac surgery	2(14)	10 (21)	0.72
Vascular surgery	0	7 (15)	0.33
Respiratory failure	9 (64)	13 (27)	0.02
Post-CPR	1 (7)	9 (19)	0.43
Shock	3 (21)	2 (4)	0.07
Sepsis	4 (29)	15 (31)	1.0
Renal insufficiency	0	4 (8)	0.57
Coma	0	4 (8)	0.57
Other	3 (21)	2 (4)	0.07
Start hydrocortisone after first test			
Number	13 (93)	45 (94)	1.0
Duration, days	7 (13)	8 (8)	0.71
Fluconazole/phenytoin	2 (14)	4 (8)	0.62
Length of stay ICU, days	18 (23)	20 (24)	0.60
Mortality day 28	5 (36)	15 (31)	0.75
Mortality ICU	5 (36)	19 (40)	1.0

Data are expressed as median (interquartile range) or number (%), where appropriate; CPR, cardiopulmonary resuscitation; ICU, intensive care unit; na, not applicable. ^a^Patients may have more than one condition.

### Etomidate in sepsis and nonsepsis

Table 3 shows characteristics according to underlying condition and etomidate use. Although all patients were initially intubated and mechanically ventilated, some of them had been extubated before the ACTH test, and the time from admission to the test was shorter in etomidate than in non-etomidate intubated patients. The patients with sepsis had a lower MAP and higher norepinephrine dose at the time of the ACTH test. Etomidate was associated with a lower MAP, particularly in nonsepsis, and higher norepinephrine requirements.

**Table 3:** Sepsis and nonsepsis on ACTH test day, and etomidate, no etomidate, and other variables before the ACTH test

	**Sepsis**		**Nonsepsis**		***P***
	**Etomidate**	**No etomidate**	**Etomidate**	**No etomidate**	
	***n *= 11**	***n *= 32**	***n *= 3**	***n *= 21**	S, E, E*S
Days admission to test	1 (2)	3 (9)	1 (2)	12 (17)	0.11, < 0.001, 0.06
APACHE II	17 (8)	18 (6)	17 (10)	14 (7)	0.29, 0.56, 0.73
Focus sepsis					
Respiratory	4 (36)	18 (56)	-	-	na, 0.52, na
Abdominal	4 (36)	6 (19)	-	-	
Genitourinary	-	1 (3)	-	-	
Soft tissue	1 (9)	3 (9)	-	-	
Endocarditis	1 (9)	-	-	-	
Meningitis	-	1 (3)	-	-	
Unknown	1 (9)	3 (9)	-	-	
Bacteria involved					na, 0.59, na
Gram^+^	2	3	-	-	
Gram^-^	3	13	-	-	
Mixed and others	1	1	-	-	
Gram^- ^bacteremia		4	-	-	na, 0.83, na
Gram^+ ^bacteremia	1	3	-	-	
Mechanical ventilation	11 (100)	28 (88)	3 (100)	15 (71)	1.0, 0.98, 1.0
Renal replacement therapy	2 (18)	9 (28)	1 (33)	1 (5)	0.31, 0.54, 0.13
MAP, mm Hg	76 (17)	73 (17)	70 (14)	91 (21)	0.02, < 0.001, < 0.001
Norepinephrine	11 (100)	22 (69)	2 (67)	9 (43)	0.005, < 0.001, 0.07
Dopamine	0	2 (6)	1 (33)	3 (14)	0.15, 0.66, 0.37

Data are expressed as median (interquartile range) or number (%), where appropriate. APACHE, Acute Physiology, Age and Chronic Health Evaluation; MAP, mean arterial pressure; S, sepsis; E, etomidate; E*S, interaction between the two in generalized estimating equations; na, not applicable.

**Figure 2:**
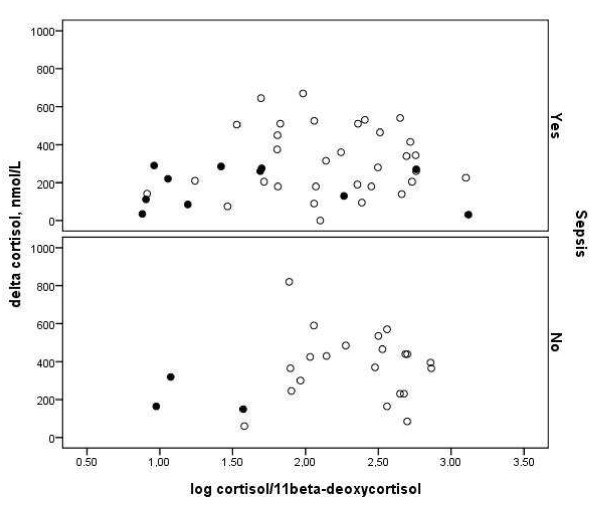
**Poor relation between cortisol/11β-deoxycortisol ratio and delta cortisol**. The figure represents the relation between cortisol/11β-deoxycortisol and the cortisol increase with exogenous ACTH, in septic (upper panel, r = 0.14; *p *= 0.77) and nonseptic (lower panel, *r *= 0.19; *p *= 0.38) patients, without (open symbols) or with (solid symbols) etomidate. For etomidate: *r *= -0.34; *p *= 0.23; for no etomidate, *r *= -0.07; *p *= 0.61.

Table 4 shows metabolic and hormonal values. Baseline cholesterol was lower in sepsis than in nonsepsis and when etomidate was used, whereas cortisol was higher in the former. The baseline 11β-deoxycortisol was elevated, and cortisol and cortisol/11β-deoxycortisol were depressed, particularly in nonsepsis, when etomidate was used. Endogenous ACTH did not differ among groups. Etomidate attenuated the increase of cortisol to exogenous ACTH in sepsis and nonsepsis alike, whereas the increase was lower in the former. Figure 2 shows that the ACTH test response did not correlate to the baseline cortisol/11β-deoxycortisol ratio in both sepsis and nonsepsis patients and whether or not etomidate was used. Indeed, the frequency of a low cortisol increase (< 250 n*M*) was not significantly affected by etomidate: for sepsis and nonsepsis together, a low cortisol increase occurred in 57% of ACTH tests preceded by etomidate and in 38% not preceded by etomidate. Six patients had etomidate with a baseline 11β-deoxycortisol > 8 n*M *and an increase in cortisol on ACTH < 250 n*M *(defining etomidate-induced adrenal dysfunction, 22), of whom four had sepsis, and three died. Finally, etomidate increased 17-OH-progesterone and decreased aldosterone relative to corticosterone, particularly in the nonsepsis group. The drug increased DHEAS and androstenedione, irrespective of the underlying condition.

**Table 4:** Metabolic and hormonal measurements on ACTH test day

	**Sepsis**		**Nonsepsis**		***P***
	**Etomidate**	**No etomidate**	**Etomidate**	**No etomidate**	
	***n *= 11**	***n *= 32**	***n *= 3**	***n *= 21**	S, E, E*S
ACTH, p*M*	1.4 (3.1)	2.4 (2.6)	1.9 (7.5)	4.1 (3.9)	0.37, 0.10, 0.22
Cholesterol, m*M *	1.3 (0.8)	1.8 (1.2)	2.4 (1.8)	2.8 (1.0)	< 0.001, 0.02, 0.75
HDL-cholesterol, m*M*	0.40 (0.40)	0.60 (0.60)	0.80 (0.80)	0.90 (0.60)	0.06, 0.18, 0.17
Progesterone, n*M*	0.40 (0.90)	0.50 (0.70)	1.0 (1.0)	0.60 (0.70)	0.55, 0.34, 0.16
Corticosterone, n*M*	3.4 (4.9)	3.5 (6.4)	5.3 (4.6)	4.0 (7.4)	0.23, 0.04, 0.64
Aldosterone, n*M*	1.3 (1.3)	0.70 (1.3)	0.50 (0.1)	1.2 (0.7)	0.08, 0.13, 0.007
17-OH-progesterone, n*M*	2.9 (2.8)	2.6 (1.5)	3.9 (2.1)	2.5 (2.4)	0.48, 0.09, < 0.001
11ß-deoxycortisol, n*M*	15 (58)	3.1 (8.3)	34 (30)	3.7 (3.3)	0.003, < 0.001, < 0.001
Cortisol, n*M*	460 (200)	500 (221)	325 (134)	455 (335)	0.03, 0.98, 0.04
Cortisol/11ß-deoxycortiso	l26 (175)	157 (352)	12 (28)	316 (380)	0.04, < 0.001, < 0.001
Cortisol/ACTH	209 (340)	223 (229)	171 (270)	109 (80)	0.07, 0.08, 0.08
Cortisol increase, n*M*	220 (190)	298 (315)	165 (169)	395 (238)	0.04, < 0.001, 0.98
Cortisol increase < 250 n*M*	6 (54)	14 (44)	2 (67)	6 (29)	0.92, 0.17, 0.43
DHEA, μ*M*	6.1 (4.6)	6.4 (4.9)	6.1 (1.8)	5.9 (4.1)	0.61, 0.61, 0.44
DHEAS, n*M*	0.70 (1.4)	0.48 (0.76)	0.50 (4.2)	0.90 (1.1)	0.39, 0.03, 0.87
Androstenedione, n*M*	3.8 (8.6)	2.4 (2.9)	3.6 (2.8)	2.8 (2.4)	0.13, 0.02, 0.60

Median (interquartile range) or number (%). ACTH, adrenocorticotropic hormone; CBG, cortisol-binding globulin; DHEA(S), dihydroxyepiandrostenedione (sulfate). S, sepsis; E, etomidate; E*S, interaction between etomidate and sepsis in generalized estimating equations; HDL, high-density lipoprotein.

### Adrenal dysfunction of CIRCI

Table 5 shows that cortisol increases < 250 m*M *were associated with low baseline cholesterol, largely independent of etomidate. Baseline ACTH was relatively high (and the cortisol/ACTH ratio low) when the increase in cortisol on exogenous ACTH was low. Etomidate decreased endogenous ACTH and increased cortisol/ACTH, particularly in the group normally responding to exogenous ACTH. Etomidate increased baseline 11β-deoxycortisol and decreased cortisol, cortisol/11β-deoxycortisol ratio, and cortisol increases on exogenous ACTH, whether the latter was low or high. Groups otherwise did not differ in 17-OH-progesterone, progesterone, corticosterone, aldosterone, DHEA, and androstenedione levels, whereas etomidate increased DHEAS levels.

**Table 5:** Adrenal dysfunction of critical illness-related corticosteroid insufficiency on ACTH test day

	**Cortisol increase < 250 n*M ***		**Cortisol increase ≥ 250 n*M***		***P***
	**Etomidate**	**No etomidate**	**Etomidate**	**No etomidate**	C, E, E*C
	***n *= 8**	***n *= 20**	***n *= 6**	***n *= 33**	
ACTH, p*M*	2.8 (4.6)	4.0 (11)	1.1 (1.8)	2.5 (2.6)	0.02, 0.001, 0.27
Cholesterol, m*M*	1.6 (1.5)	1.6 (1.0)	1.4 (0.7)	2.5 (1.0)	0.03, 0.06, 0.07
HDL-cholesterol, m*M*	0.60 (0.30)	0.40 (0.40)	0.40 (0.50)	0.90 (0.50)	0.09, 0.13, 0.04
Progesterone, n*M*	0.90 (1.5)	0.70 (0.80)	0.40 (0.70)	0.60 (0.60)	0.63, 0.83, 0.39
Corticosterone, n*M*	2.6 (3.1)	4.3 (8.0)	6.0 (7.2)	3.3 (6.5)	0.71, 0.71, 0.06
Aldosterone, n*M*	0.90 (1.2)	0.90 (1.3)	1.5 (1.3)	1.0 (1.3)	0.34, 0.95, 0.90
17-OH-progesterone, n*M*	3.2 (2.3)	2.5 (2.4)	2.5 (3.2)	2.6 (1.5)	0.10, 0.16, 0.12
11ß-deoxycortisol, n*M*	25 (56)	2.8 (11)	17 (45)	2.0 (4.9)	0.26, 0.05, 0.50
Cortisol, n*M*	427 (192)	518 (294)	460 (230)	460 (310)	0.64, 0.70, 0.06
Cortisol/11ß-deoxycortisol	13 (139)	177 (400)	38 (170)	229 (372)	0.15, < 0.001, 0.96
Cortisol/ACTH	180 (120)	107 (154)	395 (376)	165 (227)	< 0.001, < 0.001, 0.004
Cortisol increase, n*M*	121 (114)	180 (118)	280 (30)	440 (163)	na, < 0.001, na
DHEA, μ*M*	6.4 (2.6)	6.1 (5.6)	5.5 (4.1)	6.1 (3.9)	0.88, 0.48, 0.80
DHEAS, n*M*	1.0 (2.5)	0.48 (1.1)	0.55 (0.98)	0.59 (0.90)	0.55, 0.03, 0.16
Androstenedione, n*M*	3.7 (8.7)	2.9 (3.8)	3.6 (5.6)	2.2 (2.4)	0.23, 0.93, 0.19

Median (interquartile range). ACTH, adrenocorticotropic hormone; C, low cortisol increase; CBG, cortisol-binding globulin; DHEA(S), dihydroxyepiandrostenedione (sulfate); E, etomidate; E*C, interaction between the two in generalized estimating equations; HDL, high-density lipoprotein; na, not applicable.

### Multivariable models

The time from admission to ACTH test did not contribute to the following models. Baseline cortisol related to sepsis and the ACTH level (both *P *= 0.03), whereas the APACHE II score and use of etomidate did not contribute. The increase in cortisol with exogenous ACTH inversely related to baseline cortisol/ACTH ratio or ACTH (*P *= 0.01) and etomidate (*P *= 0.05) and directly to HDL-cholesterol (*P *= 0.03), but not to sepsis, baseline cholesterol, and cortisol (to 11β-deoxycortisol ratio).

## Discussion

Our study on steroidogenesis suggests that, in critically ill sepsis patients, the 11β-hydroxylase inhibiting effect of etomidate did not attenuate a relatively high baseline, stress-induced cortisol level, dissociated from endogenous ACTH. However, etomidate lowered baseline cortisol in nonsepsis, and attenuated, in both conditions, the exogenous ACTH-induced cortisol increase. Nevertheless, the effects on ACTH tests were not large enough to confound significantly the assessment of the adrenal dysfunction of CIRCI, as defined before [2,3,5,6,8-11,15,24,32]. Although etomidate thus affects exogenous ACTH-stimulated more than basal cortisol synthesis, it only partially contributes, on top of substrate deficiency, to the adrenal dysfunction of CIRCI, particularly in sepsis.

Our data suggest that baseline cortisol increased in parallel to endogenous ACTH and was therefore higher in sepsis than in nonsepsis, independent of etomidate and low (HDL-)cholesterol levels, which serve as substrates for steroidogenesis [11,15,23]. A lower cholesterol in etomidate versus nonetomidate-intubated patients can be explained in part by greater acuity of disease [15], because the time from admission to the ACTH test was shorter in the former. Otherwise, the tendency for a high cortisol/ACTH ratio in sepsis might be attributed to non-ACTH stimulation contributing to the commonly observed cortisol/ACTH dissociation [2,3,6,7,20]. In any case, the adrenals were not sensitized to ACTH because of the lower increase in cortisol with exogenous ACTH in sepsis than in nonsepsis. Our data suggest a greater effect of etomidate on baseline cortisol/11β-deoxycortisol ratio and cortisol in nonsepsis than in sepsis, whereas the effect on 11β-deoxycortisol and its ratio to cortisol reflecting 11β-hydroxylase inhibition is in line with the literature [3,4,7,16-20,22,23,26,27]. The observation that etomidate barely affected baseline HPA-axis activity, independent of the time between admission and etomidate administration and conversely on blood sampling, is supported by the lack of increase in endogenous ACTH with the decrease in the cortisol/11β-deoxycortisol ratio. Because of decreased endogenous ACTH levels with prior etomidate both in low and normal increases to exogenous ACTH, we cannot, however, exclude a small and negative effect of etomidate on the pituitary, in contrast to *in vitro *observations [17], and possibly caused by sedation and reduction of stress. The limited effect of a single dose of etomidate on baseline cortisol may relate in part to the time window chosen, because inhibition is highest shortly after administration and usually resolves after 48 to 72 hours [8,16,22,23,27-29].

In contrast to relatively spared baseline HPA axis activity, etomidate-induced inhibition of 11β-hydroxylase, which was greater in nonsepsis than in sepsis, was associated with a inhibition of the cortisol response to supraphysiologic doses of exogenous ACTH, regardless of underlying condition and cortisol-binding molecules that hardly affect ACTH-induced cortisol increases [11]. Conversely, the higher endogenous ACTH level at < 250 cortisol increases with exogenous ACTH, independent of etomidate, supports relatively insufficient baseline and exogenous ACTH-stimulated secretion by the adrenals at this cutoff, and the combination thus suggests adrenal dysfunction in the course of CIRCI, as argued before [2,3,5,6,8,10,15,24,29,32]. Conversely, the occurrence of low cortisol increases was nonsignificantly increased by etomidate, which inhibited both low and normal cortisol increases to exogenous ACTH. The level did not exceed 8 n*M *in 57% of patients with an ACTH-induced increase in cortisol < 250 n*M*, thereby supporting that etomidate and 11β-hydroxylase inhibition were not the only factors inhibiting cortisol increases. Also, the increase in cortisol with exogenous ACTH being dependent on both prior etomidate and baseline ACTH in multivariable analysis strongly supports that a low cortisol increase reflects CIRCI-associated adrenal dysfunction, partly independent of etomidate (Figure 2). This supports direct inhibition of cortisol synthesis in the adrenals, as demonstrated in septic conditions, for instance, after a substrate deficit by low cholesterol levels [2,3,6,12-14,20]. In any case, lack of evidence of inhibition of 21-hydroxylase (and 3-hydroxysteroid dehydrogenase) activity in adrenal dysfunction concords with a pediatric study on meningococcemia [20].

However, numbers may have been too low for a 19% increased risk by etomidate of low cortisol increases with exogenous ACTH to reach statistical significance, in contrast to what has been described before [4,10,16,19,22,24,28,32]. In the latter studies, greater inhibition by etomidate of cortisol increases to exogenous ACTH may have resulted in part from lower stress and cortisol baseline values and thus greater reserve on stimulation [29]. Also, effects of etomidate may be dose dependent, so that greater and more frequent inhibition of ACTH-induced cortisol increases than observed in our study may partly relate to higher doses used by others [16,17,19,24].

Etomidate did not affect circulating levels of other adrenal hormones, synthesized independent of 11β-hydroxylase. Etomidate slightly increased corticosterone relative to aldosterone (in nonsepsis), but our data do not suggest hypoaldosteronism caused by etomidate, even at low cortisol increases to ACTH, in contrast to the literature [16,18,19]. Lower MAP with prior etomidate at the time of the ACTH test, particularly in nonsepsis patients, may thus relate, at least in part, to CIRCI rather than to hyperreninemic hypoaldosteronism after a shift from mineralo- to glucocorticosteroid production [16,26,30,31], although an effect of etomidate on MAP has been refuted [29]. The increase in DHEAS (but not DHEA), which is usually low in critically ill patients, with prior etomidate treatment may reflect a shift, by diminished 11β-hydroxylase, from corticosteroid to androgen synthesis [14,34,35]. Low DHEAS and androstenedione levels otherwise support an underlying shift from androgens to corticosteroids during critical illness [26,34,35].

Our relatively small study was not designed to evaluate the effect of etomidate and replacement doses of hydrocortisone on outcome, and was therefore underpowered to judge those effects. We cannot exclude, however, that hydrocortisone-replacement therapy had offset a detrimental effect of etomidate [5,16,21,23,25,28], although some literature suggests otherwise [10,32,33]. Unchanged outcome by etomidate, in line with the literature [21,24-26], however, did not prompt us to change current practice and to abandon single doses of the drug, as suggested elsewhere [28,33]. Finally, results should be interpreted with caution because the study was not randomized, even though etomidate and nonetomidate groups were fairly comparable.

## Conclusions

Overall, our data suggest that the contribution of a single dose of etomidate, inhibiting 11β-hydroxylase up to 72 hours after its administration, on top of substrate deficiency, in the adrenal dysfunction of CIRCI is limited, particularly in stressed sepsis patients.

## Key messages

• The 11β-hydroxylase inhibiting effect of etomidate does not attenuate a relatively high baseline cortisol after stress-induced secretion by the adrenals, so that the effect on the baseline cortisol/11β-deoxycortisol ratio and cortisol is less in sepsis than in nonsepsis.

• Etomidate only partially contributes to the adrenal dysfunction of CIRCI, as assessed with the ACTH test.

• Our study confirms a primary role for a substrate deficit and low (HDL-)cholesterol levels in the adrenal dysfunction of CIRCI.

## Abbreviations

ACTH: adrenocorticotropic hormone; APACHE: Acute Physiology, Age and Chronic Health Evaluation; CBG: cortisol-binding globulin; CIRCI: critical illness-related corticosteroid insufficiency; CPR: cardiopulmonary resuscitation; DHEA(S): dehydroepiandrosterone (sulfate); GEE: generalized estimating equation; HDL: high-density lipoprotein; HPLC: high-performance liquid chromatography; ICU: intensive care unit; LC-MS: liquid chromatography-mass spectrometry; MAP: mean arterial pressure.

## Competing interests

The authors declare that they have no competing interests.

## Authors' contributions

NM and RMB participated in the design of the study, data acquisition, analysis, and drafting the manuscript. AB conceived of the study, participated in its design, and helped to draft the manuscript. CMH participated in its design and carried out the modified tandem mass spectrometry. IV and GSV participated in the design of the study and the modified tandem mass spectrometry. MFCJ participated in its design and data acquisition. ARJG participated in the design of the study. ABJG conceived of the study, carried out the coordination, participated in the design, performed the statistical analysis, and drafted the manuscript. All authors read and approved the final manuscript.
